# Barriers and Facilitators to User Engagement and Moderation for Web-Based Peer Support Among Young People: Qualitative Study Using the Behavior Change Wheel Framework

**DOI:** 10.2196/64097

**Published:** 2025-02-05

**Authors:** Ananya Ananya, Janina Tuuli, Rachel Perowne, Leslie Morrison Gutman

**Affiliations:** 1 Department of Clinical, Educational and Health Psychology Faculty of Brain Sciences University College London London United Kingdom

**Keywords:** internet, moderation, engagement, youth, teenager, adolescent, peer support, web-based group, user engagement, support group, barrier, facilitator, Theoretical Domains Framework, Behavior Change Wheel, qualitative, interview, behavior change technique, thematic analysis

## Abstract

**Background:**

Peer support groups or web-based chats for young people offer anonymous peer support in judgment-free spaces, where users may share their thoughts and feelings with others who may have experienced similar situations. User engagement is crucial for effective web-based peer support; however, levels of engagement vary. While moderation of peer support groups can have a positive impact on the engagement of young people, effective moderation can be challenging to implement.

**Objective:**

This study aimed to identify barriers and facilitators to user engagement with, and moderation of, web-based peer support groups among young people aged 16 to 25 years and to provide recommendations for enhancing this service.

**Methods:**

Drawing upon the Theoretical Domains Framework (TDF) and the Behavior Change Wheel (BCW), this study conducted qualitative interviews and gathered open-ended questionnaires from service users and moderators of The Mix, the United Kingdom’s leading web-based mental health platform providing peer support groups for young people. Semistructured interviews were conducted with 2 service users and 8 moderators, and open-ended questionnaires were completed by 7 service users. Themes were coded using the Capability, Opportunity, Motivation, and Behavior (COM-B) model and the TDF. The BCW tools were then used to identify relevant behavior change techniques to improve user engagement in, and moderation of, the service.

**Results:**

Thematic analysis revealed a total of 20 inductive themes within 10 TDF domains—9 (45%) for engagement and 11 (55%) for moderation. Of these 20 themes, 3 (15%) were facilitators of engagement, 7 (35%) were facilitators of moderation, 4 (20%) were barriers to moderation, and 6 (30%) barriers to engagement. Results suggest that skills, knowledge, beliefs about consequences, intentions, emotions, and the social and physical environment are important factors influencing service users and moderators of group chats. In particular, supporting the improvement of memory, attention, and decision-making skills of those involved; adapting the physical environment to facilitate effective interactions; and reducing negative emotions are suggested to optimize the value and effectiveness of peer support groups for young people’s mental health for both the service users and moderators of these services.

**Conclusions:**

The study demonstrates the effectiveness of the BCW approach and the use of the TDF and COM-B model to understand the influences on behavior in a systematic manner, especially for mental health and well-being interventions. The findings can be applied to design structured interventions to change behaviors related to the engagement with, and moderation of, web-based peer support groups and, in turn, improve mental health outcomes for young people.

## Introduction

### Background

Young people aged 16 to 25 years are particularly vulnerable to mental health difficulties [1]. Recent evidence shows an increase in reported mental health problems for young people aged 16 to 25 years, particularly following the COVID-19 pandemic [2,3]. For example, in young people aged 17 to 19 years in England, rates of probable mental disorders rose from 17.7% in 2020 [4] to 23.3% in 2023 [5]. Nevertheless, health care systems and support often overlook the needs of young people [6]. In the United Kingdom, 75% of young people experience delays in accessing mental health support, leading to a worsening of their condition [7].

Web-based mental health communities are a viable option to bridge the mental health service gap for young people. They offer anonymous peer support in judgment-free spaces, where users may share their thoughts and feelings with others who may have experienced similar situations [8]. Web-based peer support can be asynchronous or synchronous, providing online support in a group format, with or without moderation [9,10]. Synchronous or real-time support, such as web-based chats, provides users with in-the-moment assistance without the delays that can occur within asynchronous services [11,12]. Notably, studies have shown the efficacy of web-based peer support platforms compared to in-person talk therapies [13]. They offer clinical effectiveness [14] and scalability for public health impact, enabling outreach to a larger population [13]. Therefore, there is a strong argument for the use of web-based peer support platforms for young people as they address key barriers and hold the potential to enhance mental health treatment by providing a safe, accessible, and effective means of support.

Despite numerous benefits, studies have also highlighted challenges to web-based peer mental health support that limit its effectiveness [15], including varying engagement rates [14]. Low rates of engagement can lead to negative outcomes, such as perceived exclusion and isolation [16], whereas high rates have been found to improve young people’s mental health [17]. Effective moderation of web-based, user-led mental health services has been found to improve user engagement [18]. However, there are few studies examining user engagement in, and moderation of, synchronous web-based peer support groups, especially capturing the perspectives of both service users and moderators [11,19]. Service users, with their lived experience of giving and receiving support, can offer valuable insights and suggestions for improving these platforms. Moderators, due to their proximity to users and responsibility for supporting positive interactions, safety, and engagement [14], may have practical insights to enhance platform effectiveness [20]. A systematic examination of these behaviors from different viewpoints would enable the identification of tailored intervention strategies to improve moderation of web-based peer support services and enhance user engagement. Using the Behavior Change Wheel (BCW) [21], this qualitative research study investigates the barriers and facilitators to user engagement and moderation for web-based peer support groups among young people aged between 16 and 25 years and then proposes tailored strategies for optimization.

### User Engagement in Web-Based Peer Support

Digital health research has defined user engagement as the extent and subjective experience (characterized by attention, interest, and affect) of use [22]. Studies measure engagement in terms of use time, log-ins, or module completion [23,24], with approximately 60% of studies measuring attendance alone to assess engagement [25]. However, although passive involvement or observational participation in web-based peer support groups has recognized benefits [26], considering only attendance falls short as it omits active participation and interaction, which fosters the sense of community and support unique to this service. Therefore, this study considers engagement in the broader sense, incorporating any observable written contribution, such as comments expressing thoughts and feelings or support for others. This inclusive approach captures subjective experience, aids barrier and facilitator identification, and can inform interventions for enhanced meaningful engagement.

Previous research highlights key barriers and facilitators to young people’s engagement with digital mental health interventions and services [27,28]. Barriers include interventions that are perceived as unappealing or unhelpful, technical issues, privacy concerns, and young people lacking time or remembering to use the intervention. Facilitators include the personalization and flexibility offered by digital interventions, along with effective design, usability, opportunities to build connections, and the potential for a rewarding user experience [27,28].

However, there is a gap in research examining influences on young people’s engagement in synchronous web-based peer support groups for mental health. Barriers and facilitators are likely to differ from those in other digital mental health services, as peer-to-peer support is viewed as less stigmatizing and more relatable [29]. In addition, synchronous interactions have a different pace compared to other formats [11]. Recent research focusing on peer-to-peer chats has centered on moderators’ roles without including service user voice [30].

### Moderation of Web-Based Peer Support

Moderation involves managing content, safeguarding, and providing support to users when needed [18]. It can be carried out by health professionals or trained volunteers [19]. Effective moderation has been found to have a positive effect on user engagement and has also been helpful in preventing “toxic” web-based settings [18]. Negative effects of web-based platforms such as trolling and stalking may also be prevented by effective moderation [16]. In web-based chat groups and discussion forums, moderators ensure adherence to group guidelines to enhance user safety and prevent service users from becoming overly dependent on one another [31,32].

There has been limited research on the moderation of web-based mental health interventions [19]. Synchronous group settings pose distinct challenges as they require real-time monitoring of the chat and interaction between peers and moderators. In a recent study, Deng et al [30] explored moderation of peer support for a web-based mental health community. In this study, although moderators were qualified mental health professionals, they experienced challenges, such as dealing with emotionally triggering problems or hostile users. In a similar environment, Saha et al [33] highlighted challenges, such as a lack of concern for moderators’ well-being and moderators’ uncertainty about when to intervene. However, the services examined in these studies were not specifically designed for young people. Thus, there is a need to understand moderation and, importantly, how to enhance it, particularly in the context of real-time group chats for young people moderated by young volunteers, in a way that is structured and rooted in theory.

To address these research gaps, this study triangulates moderator and service user perspectives using a theoretically based behavior change approach, providing a comprehensive examination of user engagement and the moderation process in synchronous web-based mental health peer chats. The BCW provides a systematic and comprehensive framework to enhance understanding of behavior and support the identification of linked, relevant behavior change techniques (BCTs) to address the identified barriers and optimize group chat engagement [21].

### BCW Framework

**Figure 1:**
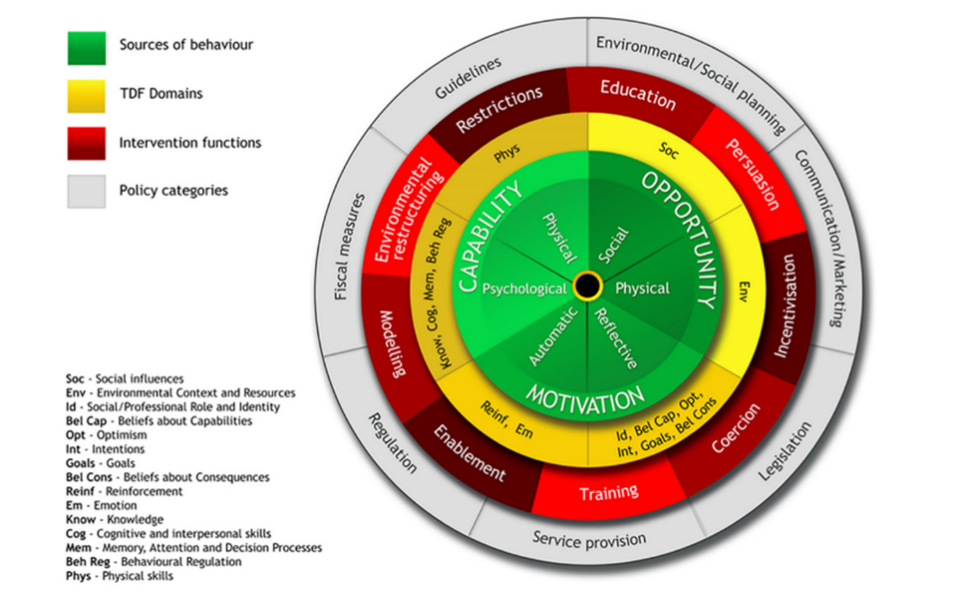
The Behavior Change Wheel and Theoretical Domains Framework (TDF) (adapted from Michie et al [21]).

The BCW comprises interconnected tools to guide decision-making and facilitate the systematic development and evaluation of behavioral interventions [21]. At its center lies the capability, opportunity, motivation, and behavior (COM-B) model of behavior, which supports the identification of the barriers and facilitators to a behavior (Figure 1 [21]). Capability refers to an individual’s physical as well as psychological capacity to perform a specific activity. Motivation is the cognitive processes that drive human behavior. Finally, opportunity refers to external factors that are outside the individual and may facilitate behavior.

The components of COM-B can be further elaborated into 14 domains using the Theoretical Domains Framework (TDF), providing a more granular understanding of the influences on behavior [34,35]. In the next layer of the BCW are intervention functions, which are the broad categories of means by which an intervention can change behavior. The outer layer consists of policy categories to support long-term, system-wide implementation.

The Behavior Change Techniques Taxonomy (BCTT) comprises 93 observable and replicable BCTs. The BCTT is a reliable method for specifying, interpreting, and implementing BCTs, which are considered “active ingredients” to facilitate behavior change either alone or in combination [36]. Expert consensus allows for mapping of the COM-B and TDF domains to intervention functions and corresponding BCTs, guiding appropriate strategies for change [37].

The BCW approach has been successfully applied in previous research to understand the barriers and facilitators to the use and delivery of digital mental health platforms for young people, including webchat counseling [28,38] and moderation of self-harm content in web-based discussion boards [39].

### This Study

Given the lack of research in this area, this qualitative study systematically explores the barriers and facilitators to the user engagement and moderation of synchronous web-based peer group chats for young people and proposes evidence-based strategies for improvement. Data were gathered from open-ended questionnaires and in-depth, semistructured interviews with young service users and moderators of The Mix, a web-based mental health support service for young people aged <25 years. Using the BCW [21], the following research questions were addressed:

1. Using the COM-B and TDF, what are the barriers and facilitators to young people’s engagement in web-based peer support groups?
2. Using the COM-B and TDF, what are the barriers and facilitators to moderation of web-based peer support groups?
3. Using the BCTT, what strategies can be used to improve user engagement in, and moderation of, web-based peer support groups for young people’s mental health?

## Methods

### Participants

This study collected data from service users and moderators of The Mix, the United Kingdom’s leading digital mental health support service for young people aged <25 years. The Mix offers free mental health support, including a helpline, phone and webchat counseling, crisis support, discussion boards, and moderated live group chats. These synchronous group chats are freely available for young people aged 13 to 25 years. Table 1 summarizes the peer-to-peer mental health group chats of The Mix analyzed in this study.

The Mix circulated a poster to moderators (total population of 52) and service users to promote participation. Interested participants contacted the researcher through email, and their involvement was entirely voluntary. As shown in Table 2, a total of 2 service users were interviewed, and a further 7 service users completed an open-ended questionnaire about their engagement. A total of 8 moderators (n=5, 63% staff and n=3, 38% volunteers) participated in interviews about moderation. Of these 8 moderators, 4 (50%) staff members and 1 (13%) volunteer also completed a separate interview about user engagement. As this was a qualitative study, it aimed to gather rich and detailed data from participants, similar to previous studies in a similar context [38,39], rather than to achieve a representative sample.

All moderators were women, while most of the service users were either women or did not self-identify their gender, with the exception of 1 man and 1 nonbinary service user. Moderators were aged between 19 and 30 years, and service users were aged between 18 and 24 years (Table 2). Moderators were involved with The Mix ranging from 6 months to as long as 8 years and worked for 1 to 2 hours per week.

Semistructured interviews, based on the TDF, were conducted via the Microsoft Teams platform and transcribed for analysis. The interviews lasted between 30 to 60 minutes each and were designed to gain in-depth service user and moderator insights into experiences of group chats regarding peer interactions, structure of the chats, and feelings about contributing. Questions included, for example, “How long do you spend on The Mix’s chat per week?” and “How do other people in the group chat make it easier or more difficult for you to share your thoughts, feelings and experiences or support others with theirs?” Questions for moderators only included the following: “How do other people influence how you contribute to the moderation of posts on the group chats?”

For user engagement only, participants were offered the alternative option to complete an open-ended Microsoft Forms questionnaire to combat any difficulties young people felt in expressing themselves in an interview and may have hindered participation [28]. The questionnaire included 5 open-ended questions, exploring what made it easier or harder for users to share their experiences, what influenced their decision to participate or not, and what changes they would make to the chat if given the opportunity.

**Table 1:** The Mix’s peer-to-peer mental health chat groups.

Group chat type	Description	Participants, n	Structure	Participation	Privacy	Moderation
Support chat	Peer support for mental health struggles	Variable	Open discussions	Active participation	No sign-up required, no password protection	Moderated by trained young volunteers and staff
Support circle	More intimate setting, where everyone takes turns to obtain support while the rest of the group listens	≤5	Rotating support and sharing	Sign up to receive support or as a “listener”	Password protected, full session attendance	Moderated by trained young volunteers and staff

**Table 2:** Participant information.

Participant	Data	Age (y)	Gender
SU1^a^	Interview	24	Man
SU2	Interview	18	Nonbinary
SU3	Questionnaire	18-25	Did not self-identify
SU4	Questionnaire	18-25	Did not self-identify
SU5	Questionnaire	18-25	Woman
SU6	Questionnaire	18-25	Woman
SU7	Questionnaire	18-25	Did not self-identify
SU8	Questionnaire	18-25	Did not self-identify
SU9	Questionnaire	18-25	Did not self-identify
MS1^b^	Interview (engagement and moderation)	28	Woman
MS2	Interview (engagement and moderation)	30	Woman
MS3	Interview (engagement and moderation)	24	Woman
MV4^c^	Interview (moderation)	26	Woman
MV5	Interview (engagement and moderation)	24	Woman
MV6	Interview (moderation)	19	Woman
MS7	Interview (moderation)	23	Woman
MS8	Interview (moderation)	22	Woman

^a^SU: service user.

^c^MS: moderator staff.

^d^MV: moderator volunteer.

### Data Analysis

Data were thematically analyzed using the 6-phase process developed by Braun and Clarke [40]. NVivo (Lumivero) and Excel (Microsoft) were used as analytical tools. The initial stage involved gaining familiarity with the transcribed interviews. A deductive coding approach was adopted initially, which involved reviewing items of data from the transcripts and questionnaires and organizing them into the TDF domains, providing a *“*start list*”* [41,42] of themes to be categorized as barriers or facilitators. The process was repeated using an inductive analysis to generate specific subthemes within each TDF theme. These subthemes were then reviewed, defined, and included in the codebook [43]. A codebook was developed to facilitate coding and was iteratively updated. As new data were added to TDF domains, facilitators and barriers were modified, expanded, or recategorized using the constant comparison method to identify patterns and variations within the dataset [44]. Triangulation was completed with data from both service users and moderators to explore consistencies and contradictions in influences on young people’s engagement with group chats. Reflexivity was prioritized to ensure openness and challenge in interpretations [45]. To ensure coding reliability and validity, 2 researchers independently cross-coded 1 transcript; a reliability check was conducted, and agreement was reached on any discrepancies.

Following data analyses, the coded TDF barriers were mapped to corresponding intervention types, using the BCW, and then specific BCTs were selected using the links specified in Cane et al [46] as well as the more recently developed Theory and Techniques Tool [47,48]. The selection of the relevant and appropriate intervention types and BCTs was informed by an appraisal of the affordability, practicability, effectiveness and cost-effectiveness, acceptability, side effects, and equity criteria [21]. This approach has been used in a multitude of studies [49,50] to evaluate intervention strategies. The selection of policy options was out of scope because the study was focused on a single organization rather than system level. Finally, the BCTs were operationalized, and intervention strategies were proposed based on a review of previous literature.

### Ethical Considerations

Low-risk ethics approval was obtained from the University College London Ethics Committee (Z6364106/2023/03/149 social research, 25069/001). Participant information and a consent form were provided to interested parties to sign digitally and confirm consent. Participants were advised that participation was completely voluntary and they could withdraw at any time, up to 4 weeks after their data were collected. As reimbursement for the interviews, participants were given a £10 (US $12.45) voucher. Questionnaire respondents could opt into a lottery prize draw to win 1 of 2 £10 (US $12.45) vouchers by providing their email address. To protect participant privacy and confidentiality, data were fully anonymized.

## Results

### Overview

Table 3 sets out the themes identified as both barriers and facilitators to user engagement and moderation. These are set out according to both COM-B and TDF frameworks.

**Table 3:** The COM-B^a^ and TDF^b^ themes and subthemes for user engagement and moderation.

COM-B and TDF themes		User engagement subthemes	Moderation subthemes
**Psychological capability**			
	Cognitive and interpersonal skills	Difficulty expressing feelings and needs (barrier) Skills to listen and validate (facilitator)	Developing skills (facilitator)
	Knowledge	—^c^	Understanding the guidelines (facilitator)
	Memory, attention, and decision-making	Memory recall of guidelines (barrier)	Responding quickly in complex situations (barrier)
	Behavioral regulation	—	Holding back on advice (barrier)
**Reflective motivation**			
	Beliefs about consequences	Judgment and confidentiality concerns (barrier)	Impact of moderation (facilitator)
	Social and professional role and identity	—	Congruence between social and professional identity (facilitator)
	Intentions	Wanting to support others (facilitator)	Intending to shape young people’s perceptions (facilitator)
**Automatic motivation**			
	Emotions	Fear and anxiety (barrier)	Distressing subject matter (barrier)
**Physical opportunity**			
	Environment, context, and resources	Overload and lack of structure (barrier)	Organizational resources (facilitator) Lack of visual cues (barrier)
**Social opportunity**			
	Social influences	Similarity and familiarity (facilitator) Integration of new users and gaps in support (barrier)	Support offered (facilitator)

^a^COM-B: Capability, Opportunity, Motivation, and Behavior.

^b^TDF: Theoretical Domains Framework.

^c^Not applicable.

### Young People’s Engagement

As shown in Table 3, a total of 9 barriers and facilitators were identified as subthemes across 7 domains of the TDF. Themes were mainly consistent across support chat and support circle responses, but a small number of differences arose between the 2, and these are indicated, where relevant, in the following subsections.

### Cognitive and Interpersonal Skills (Psychological Capability)

#### Difficulty Expressing Feelings and Needs

Several service users and moderators described the difficulty users face in expressing their emotions and thoughts, which can prevent them from opening up in the chat. Subthemes are described below, together with illustrative quotes. Service user quotes are indicated by “SUX,” volunteer moderator quotes are indicated by “MVX,” and staff moderator quotes are indicated by “MSX,” where “X” is the participant number, to identify each participant as per Table 2. One user commented the following:

[What makes it more difficult to open up is] not being able to express myself about feelings.

SU8

Moderators also noted that vague statements about one’s emotional state without specific requests for support may be a barrier to others’ engagement, as they provide insufficient guidance for other users to offer relevant support. For example, one user stated the following:

I think it depends on how someone phrases the question...if they’re sort of saying, “And I’m feeling really low today” or “And I can’t do this anymore,” like some of those trickier comments.

MV5

#### Skills to Listen and Validate

Both moderators and users identified that having skills to effectively validate and listen to others facilitated peer support and engagement. One moderator noted the following:

The people that tend to offer the most peer support are those that...know how to validate someone’s experiences.

MS1

Users described how they did this, with one user stating the following:

Everyone listened and waited their turn...I listened to others, and they encourage people waiting their turn to help others.

SU6

Personal experiences that related to others’ struggles made it easier to connect and offer support, but 1 user stated the following:

There are some situations where I can’t exactly relate...so I stay quiet.

SU5

### Memory, Attention, and Decision Processes (Psychological Capability): Memory Recall of Guidelines

Difficulties in absorbing, retaining, and applying guidelines posed a significant barrier to a couple of users. Users indicated that they struggled to remember guidelines due to content volume, distractions, and cognitive limitations, impacting their ability to engage. Even long-term users found it challenging, with 1 individual expressing the following:

I can’t actually remember what [the guidelines are]. They’re very basic from what I do remember.

SU1

Mental health crisis situations further exacerbated guideline oversight, leading some users to unintentionally deviate from the chat’s intended mode of engagement in the moment. Moderators noted that this issue was worsened by some users, particularly regular ones, who found the guidelines unengaging and tended to ignore them.

### Beliefs About Consequences (Reflective Motivation): Judgment and Confidentiality Concerns

This was a barrier revolving around young people’s apprehensions about being judged, misunderstood, or facing negative reactions when sharing personal thoughts and experiences, particularly in the presence of new members. One participant stated the following:

Some people feel like a new person may judge more or less or may be offended more or be offended less. So [withdrawing] very much sort of safeguarding themselves, protecting themselves, and protecting others.

SU1

This could also lead users “to not give too much information” (SU7). Similarly, a moderator highlighted concerns about users fearing judgment, with 1 moderator stating the following:

...judging that person for talking about that cause I think that’s probably what they worry about quite a lot.

MV5

Both moderators and users mentioned concerns about confidentiality, giving examples of where their privacy was violated by other members, leading to negative consequences. As a result, they became hesitant to share. One moderator stated the following:

Some members also worry confidentiality will be broken so they don’t open up in fear of the police being called.

MS2

### Intentions (Reflective Motivation): Wanting to Support Others

Overall, the intention to create a supportive environment was a facilitator, which motivated users to engage to support others. One user expressed the following:

Most of the time, I try my best to offer support and be there for the person too. I just want people to be able to open up to me, and when they do, I try to be a good friend.

SU2

However, moderators were concerned that some users may prioritize self-support, limiting their involvement in supporting others and welcoming newcomers. One moderator stated the following:

Often times, the young people are so busy sharing their own experiences that they don’t make enough space to support others who may be going through a similar thing.

MS1

### Emotions (Automatic Motivation): Fear and Anxiety

A frequently mentioned barrier by both users and moderators related to the user’s fear of being perceived as a burden by sharing their thoughts, feelings, and experiences. One user expressed the following:

As for not opening up so much that’s more when I feel like I’m a burden and not wanted, but that’s me not The Mix.

SU4

Users identified that “Worrying what people think” (SU7) in terms of oversharing or repeating themselves caused them to hold back or stop sharing altogether. Another user shared similar sentiments, noting that opening up more “doesn’t really make me feel better, it just makes me anxious and guilty” (SU2). These feelings of self-doubt and anxiety hindered users from fully opening up and receiving the support they needed.

### Environmental Context and Resources (Physical Opportunity): Overload and Lack of Structure

A barrier identified by almost all participants was the lack of structure and organization within the support chat, especially during busy times. This could result in confusion, overwhelm, and a sense of disorganization. Users sometimes struggled to fully engage and provide or receive support effectively because, as 1 user said, “When you have a lot of people, you do get to a point where you do have like five, ten different conversations going on, all different levels of importance so it does get a bit confusing” (SU1) and “It can be hard to type that fast” (SU6). One moderator added the following:

It can be hard for someone to post, and their message may get missed.

MV5

Another user highlighted the following:

Support Chat needs to be more organized...The way it currently is...stresses me out, I find myself leaving midway most of the time.

SU9

### Social Influences (Social Opportunity)

#### Similarity and Familiarity

The presence of individuals who had experienced similar situations or who were known to each other was highlighted by moderators and users as helping foster a sense of belonging and encouraging engagement. One user noted the following:

It helps that there are people who know what you’ve been through, so you don’t feel alone. It makes you feel more comfortable to open up.

SU1

It was seen to be “Easier to have people with similar problems and get ideas surrounding mental health teams etc.” (SU3). Several long-term users also highlighted that regular participation by both users and moderators builds trust and familiarity, facilitating engagement. One user stated the following:

Because we’ve been with [the moderators] for some time, they understand our situations a little bit more so it makes it easier for us to open up to them and...we can trust them.

SU5

Moderators shared similar insights, noting that regular users “feel quite attached to the moderators, so they’ll leave support circle and join support chat for a bit and then come back” (MV5).

#### Integration of New Users and Gaps in Support

A frequently mentioned barrier by several members and most moderators related to how some regular users tended to chat among themselves or with the moderators, making it difficult for new users to integrate into the group. This could lead to new users feeling like outsiders and being less likely to engage. One user stated the following:

When I first started coming to circle, it was very awkward for me because the other people in there had been doing it a lot longer and I felt like a bit of an outsider.

SU5

New users described not receiving responses to messages, which seemed to have a notable impact on them. One of them stated the following:

What prompts me to withdraw is when some of my messages get ignored. I know it’s not on purpose but it kind of makes me feel unwanted.

SU4

This was a theme that was also recognized by multiple moderators as a deficiency in social support.

### Moderation

As shown in Table 3, a total of 11 barriers and facilitators were identified as subthemes across 10 domains of the TDF. The following paragraphs describe the themes in more detail, along with selected quotes.

### Cognitive and Interpersonal Skills (Psychological Capability): Developing Skills

Most moderators discussed how they developed their skills over time through a continuous learning process, which helped them become more effective and refine abilities such as active listening and conflict management:

But it’s like a continuous learning process, so I wouldn’t say they give you kind of training at the beginning of you know, how to be an active listener and how to give that specific type of support.

MS1

Observing and practicing moderating also helped develop the necessary skills for moderation. One moderator expressed the following:

I think a lot of it is just sort of the exposure and just sort of the repeated attendance of chats and you sort of build, you work out sort of what things you’ve said have gone down well.

MS7

### Knowledge (Psychological Capability): Understanding the Guidelines

Knowing and understanding the guidelines and procedures laid out by The Mix was a recurrent enabling theme of moderation, mentioned by all moderators, facilitating the identification of what is and is not deemed inappropriate. One moderator expressed the following:

And then the handbook also has things around the tech side of things, so using the platform how to use the moderator functions as well, so we’re able to mute people. We’re able to freeze them and then remove them from the room as well.

MS3

The guidelines were also particularly helpful for directing individuals at risk through appropriate safeguarding procedures. One moderator stated the following:

“It’s got things from and sort of managing young people that come in who are in crisis. So many kinds of questions to ask safely within the room and the kind of signpost we can give.

MS2

### Memory, Attention, and Decision Processes (Psychological Capability): Responding Quickly in Complex Situations

The complexity of group chats acted as a barrier for most moderators, presenting challenges by their fast-paced nature and situations that moderators may not have come across before. Attentively responding to the chats becomes challenging. One moderator expressed the following:

But there’s only so fast you can type, and only so many conversations you can kind of have going on at once that you can’t talk to everybody.

MS8

In addition, dealing with novel or gray situations caused confusion about what is acceptable in the chat and what is not, making decision-making difficult. One moderator stated the following:

There’s still some situations that catch me off guard. You know, some people saying things that I’ve not come across before, experiencing things that I’ve not come across before.

MV5

### Behavioral Regulation (Psychological Capability): Holding Back on Advice

Having a natural inclination to offer advice made it difficult for some moderators to self-monitor their responses. Going against instincts and refraining from giving advice when young people shared their problems required self-monitoring of behavior. One moderator expressed the following:

It is like to go against that nature and leave it open to the room to support each other and not give advice.

MS1

As moderators found it difficult to hold back, effective moderation became challenging. One moderator stated the following:

We can’t give medical advice, it’s tough...we shouldn’t really be saying (giving advice) to someone.

MV5

### Belief About Consequences (Reflective Motivation): Impact of Moderation

Overall, the perceived outcome of moderation for young people, as understood by moderators, facilitated their moderation. Moderators had different beliefs on the impact that moderation had on young people. Some believed that moderation helped create a safe space. One moderator expressed the following:

I think young people find it really reassuring to have, like, moderators there because there’s a lot of online support spaces, but they’re not always moderated or kept safe.

MS2

Some moderators felt that young people could feel left out and unsupported. One moderator stated the following:

And sometimes the young people can feel like the moderators are not responding to them.

MS3

The vast coverage of the impact of moderation was also highlighted by moderators. One moderator expressed the following:

I think you do notice the impact because it covers everything OK, like mental health is the main domain. But it’s also like everything like education, careers, drugs and alcohol. Just like everything that someone could be going through. So I think you do notice a wider impact.

MV4

### Social or Professional Role and Identity (Reflective Motivation): Congruence Between Social and Professional Identity

The alignment of personal values and social identity with the professional role of a moderator facilitated moderation for all moderators. One moderator expressed the following:

I love volunteering and I love working in a church in the voluntary sector and working with charities. So and I like working with young people. A lot of my work has been with young people. So and yeah, I think it does. It does fit in that respect personally.

MS3

The role of a moderator aligned with the identity that moderators had built for themselves. One moderator stated the following:

I also work in mental health in my 9 to five job, so that really helps and that’s why I wanted to start it in the first place because I started the chat at university and I was trying to broaden my experience working with people with mental health conditions.

MS7

### Intentions (Reflective Motivation): Intending to Shape Young People’s Perceptions

Some moderators expressed a deliberate intention to shape and manage young people’s perceptions and expectations of the chat. One moderator expressed the following:

I’ve tried over the years to change the young peoples’ perception of what the chat should be used for, I think that in the spirit of trying to make it more of a group conversation like a group chat.

MS1

This intention served as a strong facilitator for moderators. One moderator stated the following:

So I guess how we manage those situations, yeah, we can warn community members like, we encourage them to take a step back and let the moderators manage the situation. And yeah, we always let them know like they might be removed from the room if they don’t like, listen to us.

MS2

### Emotion (Automatic Motivation): Distressing Subject Matter

Most moderators identified that topics discussed in group chats, such as self-harm and suicidal thoughts, could take an emotional toll on moderators and cause stress and exhaustion. One moderator stated the following:

The topics that are being discussed are quite distressing. Examples are feeling very, very low and sometimes it becomes a bit of an echo chamber of you know, they’re all talking about feeling like they want to end their life or feeling like self-harming.

MS1

These topics also gave way to discussions about the larger mental health ecosystem and evoked concern among the moderators for the young people. One moderator expressed the following:

Hard when you see them struggling. And so yeah, I think when that happens, you like worry quite a lot and it does kind of be on your mind for like a few days.

MS2

These distressing thoughts and related feelings inevitably acted as a barrier for moderators.

### Environmental Context and Resources (Physical Opportunity)

#### Organizational Resources

The multitude of resources provided by The Mix was appreciated by all moderators. These encompassed guidelines, debrief forms, newsletters, and more, helping them moderate effectively. One moderator stated the following:

It really helps to have that handbook there to walk you through those guidelines and how to respond.

MS3

Moderation was facilitated through the ready availability of these resources, providing moderators with content that could be used in the chat. One moderator expressed the following:

And we have our Mod handbook which is really helpful in terms of giving us like example messages to pop in the group chat and to manage certain situations.

MS7

#### Lack of Visual Cues

Working in a web-based environment meant that moderators had to work without any visual cues, such as facial expressions. One moderator stated the following:

I think moderating is quite a unique task...especially when it’s online.[MS2]

This absence made it difficult for moderators to gauge the intensity of young people’s emotions and thus required extra effort and attention, which could be a barrier for some moderators. One moderator expressed the following:

I guess you have to put a bit of extra effort into that because you don’t have the visual cues of like, nodding and all that stuff.

MS1

### Social Influences (Social Opportunity): Support Offered

Support offered during and after moderation (eg, support from supervisors and emails checking up on moderators) acted as an enabling influence. All respondents had an overwhelmingly positive response toward the support that they received from their supervisors and believed that this support helped them moderate. A respondent stated the following:

[The Mix] never makes you feel unappreciated. Like we get quite regular emails of just a reminder that you’re all doing really good things...and I think yeah, when you have had a difficult chat and sort of things, they’re feeling a bit hard, just things like that really make a difference.

MS7

In addition, volunteers reported that the presence of a supervisor in the chats made moderation easier. One moderator stated the following:

The moderator would flag it to the supervisor on shift and just say you know ohh it looks like these two are having a bit of a disagreement. What should we do?

MS1

One moderator expressed the following:

They are really understanding about wanting someone else to step in with a conversation that hits too close to home or needing 5 mins to yourself during the session. There is also a debrief form and if I say anything I felt unhappy with, they always chase it up to check on me or ask if I want more training in the area.

MV6

### BCTs to Address Barriers

To optimize user engagement, Table 4 presents the BCTs identified to address the core barriers and enhance the facilitators, along with examples.

Table 5 shows the potential intervention types along with the corresponding BCTs to overcome the identified barriers to moderation.

**Table 4:** Barriers to engagement and strategies for change.

TDF^a^ domain	Barrier	Intervention types	Potential BCTs^b^	Operationalization of BCTs
Cognitive and interpersonal skills	Difficulty expressing feelings and needs	Training	Instruction on how to perform a behavior (4.1^c^); behavioral practice/rehearsal (8.1)	Self-reflection worksheets: provide optional worksheets with guidance on identifying emotions and the support needed before users’ participation in the group chat.
Memory, attention, and decision processes	Memory recall of guidelines	Enablement	Commitment (1.9)	Group agreement (in the support chat): collectively agree to abide by principles and behaviors aligned with the guidelines at the start of the support chat.
Memory, attention, and decision processes	Memory recall of guidelines	Education	Prompts/cues (7.1)	Reminders: implement periodic reminders or prompts about the chat guidelines to reinforce their importance and improve memory recall.
Beliefs about consequences	Judgment and confidentiality concerns	Education	Information about emotional consequences (5.6)	Share success stories: highlight success stories or testimonials from other young individuals who have benefited from engaging in peer-to-peer group chats.
Emotions	Fear and anxiety	Enablement	Reduce negative emotions (11.2)	Clarify burden misconceptions: provide information and resources from peers about the value of sharing and supporting each other, and share examples of challenges discussed by previous service-users in groups chats to address misconceptions that sharing is a burden and facilitate engagement.
Environmental context and resources	Overload and lack of structure	Environmental restructuring	Restructuring the physical environment (12.1)	Group similar topics and establish topic rotation: encourage moderators and members to brainstorm and group similar topics together either during the support chat (eg, via a poll website such as Slido) or before the chat (eg, via a poll on the discussion boards), allowing for more focused and meaningful discussions instead of fragmented conversations (alternative: create more themed chat sessions).
Environmental context and resources	Overload and lack of structure	Environmental restructuring	Restructuring the social environment (12.2)	Limit chat size: implement a cap on the number of members allowed in the support chat at 1 time to maintain a manageable and supportive group size.
Environmental context and resources	Overload and lack of structure	Environmental restructuring	Adding objects to the environment (12.5)	Emoticons: consider moving to a new chat software that enables members to offer immediate reactions to others’ messages via emoticons rather than messages.
Social influences	Integration of new users and gaps in support	Enablement	Social support (unspecified; 3.1) and social support (practical; 3.2)	Buddy system: assign new users a designated “welcoming buddy” (peer) whose role is to encourage or practically facilitate interactions during their initial sessions; grouping or pairing system: pairing or grouping members in the chat as each other’s dedicated “supporters” for the session to enhance peer support and encourage more engagement.

^a^TDF: Theoretical Domains Framework.

^b^BCT: behavior change technique.

^c^Behaviour Change Technique code numbers as per the Behaviour Change Technique Taxonomy provided in Michie et al [21].

**Table 5:** Barriers to moderation and strategies for change.

TDF^a^ domain	Barrier	Intervention types	Potential BCTs^b^	Operationalization of BCTs
Memory, attention, and decision processes	Responding quickly to complex situations	Training	Behavioral practice/rehearsal (8.1^c^)	Practice sessions: create practice moderation sessions that simulate the quick nature of group chats, which could help moderators to enhance their attention and decision-making processes.
Behavioral regulation	Holding back on advice	Training	Self-monitoring of behavior (2.3)	Diaries: ask moderators to make a note of situations and monitor when they feel instinctively inclined to offer advice to avoid it in the future.
Emotion	Distressing subject matter	Enablement	Reduce negative emotions (11.2)	Positive messaging: providing information about the positive impact of moderation through regular updates (eg, weekly or biweekly emails such as “Here’s the impact that you helped deliver!”) to increase moderators’ positive emotions and reduce concerns about young people.
Environmental context and resources	Lack of visual cues	Environmental restructuring	Prompts/cues (7.1)	Phrase book: prompts or cues may be provided to compensate for the lack of visual cues. A booklet of phrases or “words to look out for” may be provided such that moderators may look out for these words to gauge if an individual is at risk, which may otherwise be missed.

^a^TDF: Theoretical Domains Framework.

^b^BCT: behavior change technique.

^c^Behaviour Change Technique code numbers as per the Behaviour Change Technique Taxonomy provided in Michie et al [21].

## Discussion

### Principal Findings

This study addresses a research gap by investigating the influences on engagement with, and moderation of, synchronous web-based peer support group chats to support young people’s mental health. Using the BCW framework, thematic analysis revealed a total of 20 themes, 9 (45%) for engagement and 11 (55%) for moderation. Of the 20 themes, 3 (15%) were facilitators of engagement, 7 (35%) were facilitators of moderation, 4 (20%) were barriers to moderation, and 6 (30%) were barriers to engagement. The following discussion focuses on the COM-B and TDF themes that were prominent and common across both user engagement and moderation. The findings of this study are discussed in relation to previous research, and the potential BCTs to address the identified barriers to engagement and moderation are contextualized.

### Facilitators Common to Moderation and Engagement

Cognitive and interpersonal skills and knowledge (psychological capability) enabled users to express their feelings and needs. Both moderators and users benefitted from having the skills to listen and respond to other users. Moderators developed these skills through practice and were supported by a strong knowledge and understanding of the chat guidelines. Similar themes have been found in other modes of web-based mental health support [39].

Intentions (reflective motivation) were also core themes for moderators and users—both were driven by their desire to help others and ensure that the chat was a valuable resource for users. This sentiment is echoed elsewhere in the literature on web-based support groups, where listeners aimed to create a *“*safe and warm” space for their clients [51]. Similarly, moderators in this study consciously resolved to provide a safe and nonjudgmental space for young people. This is a theme that can be seen elsewhere in the literature, relating to other web-based peer mental health communities [30].

Finally, for both engagement and moderation, social influences (social opportunity) was another facilitator, where other users, moderators, or supervisors were seen as understanding, supportive, and appreciative of others’ needs. For users, this meant being able to share experiences in a safe environment, and moderators felt valued, appreciated, and encouraged in their moderation. Such support also promotes moderators’ mental health. As has been previously suggested by Aldamman et al [52], perceived organizational support was positively related to mental well-being, reduced emotional exhaustion, and reduced stress among humanitarian volunteers. This aspect is also linked to the potential of moderators to contribute to the enhancement of the mental well-being of service users, as demonstrated by Perry et al [19].

### Barriers Common to Moderation and Engagement

Group chats present a complex environment due to their dynamic and fast-paced nature, with multiple users. Users could find it difficult to remember and adhere to the guidelines, especially in pressurized situations (eg, crises). Although moderators were familiar with the guidelines, they found it challenging to make real-time decisions, particularly during busy chats when many users were interacting rapidly with one another. This inherent complexity can function as a barrier to effective moderation and engagement. With a continuous inflow of new messages in group chats, it becomes challenging to focus on messages and identify any specific theme, as noted by Li et al [53]. Moderators in this study also encountered difficulties in promptly and accurately assessing messages for potential at-risk situations, under time pressure, while simultaneously ensuring that all the young people in the chat felt supported. The BCT of “Behavioral practice” can help equip moderators to handle novel situations quickly as they arise. This BCT has been successfully used to “positively influence” behavior in the context of user training [54] and in interventions supporting shared decision-making [55].

The challenges with rapid decision-making of how to respond partly resulted from the chat environment being unstructured and uncontrolled in terms of the volume and speed of message exchanges; this was a barrier for moderators and users. Implementing emoticons (BCT “adding objects to the environment”) could offer an alternative and rapid way of engaging with messages, helping to express emotions while reducing message overload [56]. Previous studies have also highlighted participants of a Cognitive Behavioral Therapy-based peer support platform feeling overwhelmed and stressed due to message volume and unfamiliar dynamics [57]. This underscores the need for accessible and well-organized chat platforms. One promising BCT is “restructuring the physical environment.” Similar topics could be grouped within the platform or support chat sessions could be themed to allow users to explore and self-organize into groups based on shared characteristics and pain points, as has been implemented elsewhere [58].

Another aspect of the environment that was highlighted in previous studies was the lack of visual clues to support decision-making. This was a particular barrier for moderators, impeding connection and relationship building [39,59]. The BCT “prompts/cues” has been previously suggested in a similar context to improve moderation [39]. These could be in the form of electronic prompts or suggested phrases that pop-up when a young person writes a phrase that may need to be flagged. Such prompts may support moderators to identify individuals at risk and compensate for the lack of visual cues. This BCT has been successfully used to prompt action by the user in various contexts [60] and can also be used to simultaneously address barriers related to memory difficulties.

Given the sensitive nature of the discussions within web-based peer support chats, it is perhaps unsurprising that emotions can constitute another barrier. The BCT of “reduce negative emotions” can help address this. For users, the fear and anxiety associated with disclosure can prevent them from sharing in the chats, hindering their access to the help they need. Previous interventions report participants finding it helpful to know that others were undergoing similar emotions, reducing feelings of isolation [61]. Therefore, providing information and resources from peers about the value of sharing and supporting each other, and sharing examples of challenges discussed in group chats by previous service users may facilitate engagement. This could further build a sense of a supportive and friendly community on the platform, which facilitates willingness to share feelings and difficulties in other contexts [57]. Dealing with distressing topics such as mental health issues, suicidal thoughts, and other issues that young people are dealing with often becomes emotionally exhausting for moderators. In this case, the BCT “reduce negative emotions” could involve providing moderators with information about the positive impact of their moderation, to counter any negative emotions. A scoping review highlighted that this BCT has frequently been used in the development of mental health interventions [62]. Resilience-building training may also be helpful in equipping moderators to deal with the “emotional cost” of content moderation [63].

### Barriers Specific to User Engagement

The barrier of “difficulties expressing feelings and support needs” experienced by users aligns with a prior study on webchat counseling engagement among young people, which found users lacking self-expression skills [28]. “Constructive emotional sharing” is a skill that can be improved with practice [64]. Promising BCTs to address this barrier include “instruction on how to perform a behavior” and “behavioral practice/rehearsal.” For example, self-reflection worksheets could encourage users to identify their feelings and practice written statements before participating in group chats [64]. Statements such as “I feel...when...because” could be shared, leading to more personalized responses [65]. These BCTs could prove particularly beneficial for newcomers or individuals less familiar with the dynamics of group chat interaction, also creating positive spillover to a further barrier “integration of new users and gaps in support.” A barrier identified here aligns with a previous study, which found that receiving empathic comments initially has a significant cascading effect, motivating individuals to reciprocate and offer support to others [66]. To further encourage integration and support for new users, the BCT “social support” could be introduced by assigning a designated “welcoming buddy” whose role is to support new members during their initial sessions or pairing members in the chat as each other’s “supporter” to enhance peer support and encourage more engagement. Prior research has shown that shared interests encouraged conversation between new pairs in a peer support intervention, who were strangers when they were initially paired [67].

Having judgment and confidentiality concerns constituted another barrier. Overcoming such concerns can contribute to more fluid communication in web-based peer support and are a core component of building trust [67]. The BCT “information about emotional consequences” may address this barrier through sharing success stories, statistics, or personal narratives. Within the recovery model, this increases a sense of acceptance, understanding, and authenticity, particularly for new service users who tend to experience these fears more than long-term users [68].

### Barriers Specific to Moderation

Moderators found it difficult to hold back on offering advice. Rooted in the logic of care [69], moderation is contextualized and involves an empathetic dialogue or interaction between moderators and users [70]. In such an open-ended process, holding back, given the natural flow of an interaction, proved a challenge for moderators. “Self-monitoring of behavior” could be a potential BCT that may help moderators to keep their behavior in check and change it. This could be achieved by encouraging moderators to note when they instinctively offer advice or feel like they want to do so, allowing them to reflect and monitor their own behavior if such situations arise again. This technique has proved effective in many behavior change interventions in different contexts [71,72], particularly for health behaviors [73,74].

### Limitations

The findings of this study need to be considered in light of certain limitations. The sampling of this study was reliant on self-selection, which may have introduced bias and favored participation by more confident users and moderators, potentially influencing the barriers and facilitators identified [75]. Although the participants were assured that their responses would remain confidential and would not have any consequences with respect to their relationship with The Mix, they may nevertheless have given socially desirable responses, whether intentionally or subconsciously [76]. Furthermore, it is noteworthy that the study participants were predominately women, making the sample relatively homogeneous. However, the overall population of moderators and group chat users at The Mix is also mainly women (>70% of users and 95% of moderators). Finally, the study’s findings are grounded in the data obtained from a single mental health group chat forum, which could limit the generalizability of the results to other chat contexts operating under different circumstances.

### Conclusions

Group chats are an increasingly popular form of digital mental health intervention, and this study contributes to building the evidence base, which can help optimize them as a safe and timely form of mental health support for young people. It is particularly valuable as it examines synchronous group chats, which are characterized by in-the-moment empathic interactions and emotional connections. Through using the COM-B and TDF, the study found that skills and knowledge, beliefs about consequences and intentions, emotions, and the social and physical environment are important factors influencing both the users and moderators of group chats. In particular, supporting the improvement of memory, attention, and decision-making skills of those involved; adapting the physical environment to facilitate effective interactions; and reducing negative emotions are suggested to optimize the value and effectiveness of group chats for young people’s mental health support for both the users and moderators of these services. The intervention types and BCTs proposed serve to emphasize the importance of training and support, particularly for moderators, in this important role. The study also further demonstrates the effectiveness of the BCW approach and the use of the TDF and COM-B to understand the influences on behavior in a systematic manner, especially for mental health and well-being interventions.

A natural progression of this work would be to implement and evaluate the interventions proposed in the study and gauge to what extent the suggested BCTs reduce the identified barriers. In addition, the fidelity of the BCTs could also be assessed to understand the nuances of intervention delivery [77] to facilitate contextualized tailoring of the intervention. In an environment where digital mental health interventions for young people continue to grow in significance, this study and future suggested studies can contribute toward ensuring that they are evidence based and consider the voices of young people themselves.

## Abbreviations

BCT: behavior change technique
BCTT: Behavior Change Techniques Taxonomy
BCW: Behavior Change Wheel
COM-B: Capability, Opportunity, Motivation, and Behavior
TDF: Theoretical Domains Framework
